# Unveiling Rarity: Giant Septal Coronary Vein Dissection, an Unforeseen Twist in Complications of Left Bundle Branch Area Pacing

**DOI:** 10.19102/icrm.2025.16022

**Published:** 2025-02-15

**Authors:** Ronpichai Chokesuwattanaskul, Monravee Tumkosit, Krit Jongnarangsin

**Affiliations:** 1 Department of Medicine, Faculty of Medicine, Cardiac Center, King Chulalongkorn Memorial Hospital, Chulalongkorn University, Thai Red Cross Society, Bangkok, Thailand; 2 Center of Excellence in Arrhythmia Research Chulalongkorn University, Department of Medicine, Faculty of Medicine, Chulalongkorn University, Bangkok, Thailand; 3 Department of Radiology, Faculty of Medicine, Chulalongkorn University, King Chulalongkorn Memorial Hospital, Bangkok, Thailand; 4 Division of Cardiac Electrophysiology, University of Michigan Health Care, Ann Arbor, MI, USA

**Keywords:** Conduction system pacing, coronary vein dissection, left bundle branch area pacing

## Abstract

Left bundle branch area pacing (LBBAP) is a promising pacing technique aimed at mitigating pacing-induced cardiomyopathy; however, a gap in understanding persists concerning intraprocedural complications and their management. This case study sheds light on a rare complication associated with LBBAP. Through sheath penetration into the interventricular septum at a typical site, a septogram revealed the dissection of a large septal coronary vein. Herein, we propose the management strategy and its outcome.

## Introduction

Left bundle branch area pacing (LBBAP) has emerged as a valuable technique for facilitating physiological pacing through the conduction system, aiming to mitigate the adverse effects associated with permanent right ventricular pacing. While complications related to coronary veins appear to be infrequent and generally benign, there is a paucity of literature addressing this issue. Moreover, there is no consensus regarding the necessity of recording a septogram during lead penetration. Potential serious complications include perforation and injury to coronary vessels. An intervention proposed for avoiding coronary venous fistula is recording a septogram to visualize small septal veins.^1^

## Case presentation

**Figure 1:**
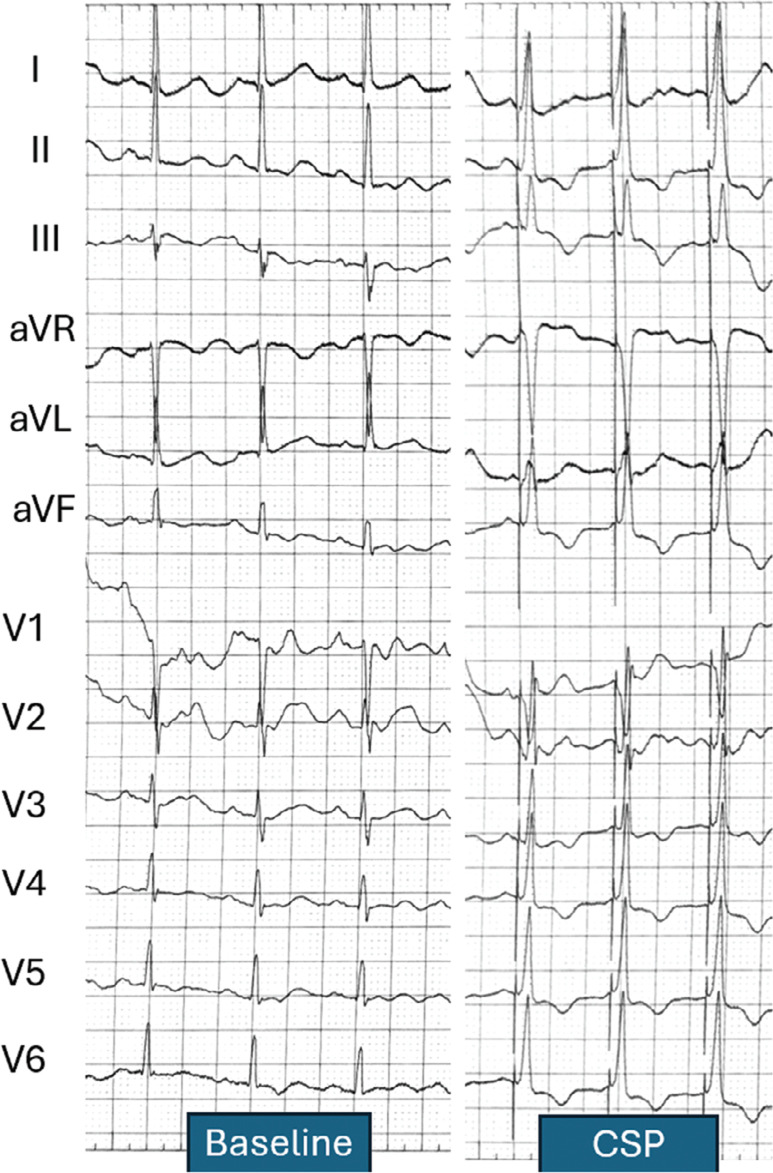
Surface electrocardiogram. First three complexes: intrinsic rhythm (normal sinus rhythm with normal P–R interval and QRS duration). Last three complexes: left bundle branch–paced QRS morphology. *Abbreviation*: CSP, conduction system pacing.

**Figure 2:**
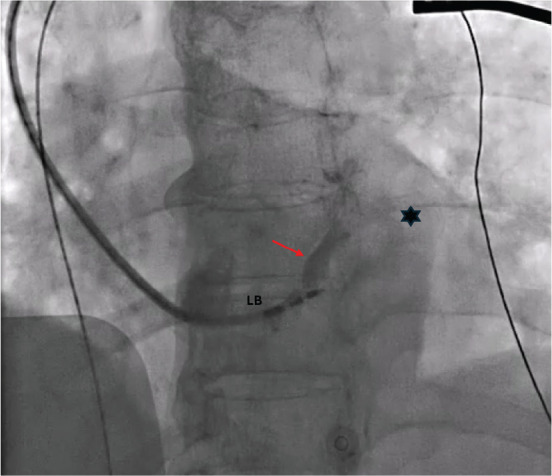
Fluoroscopy projection from the left anterior oblique angle reveals the penetration of the interventricular septum by the lead (LB), as indicated by the red arrow. The image also depicts the perforator branch vein (red arrow) and the contrasted coronary sinus (marked with an asterisk) through flow from the sheath. *Abbreviation:* LB, left bundle pacing lead.

**Figure 3:**
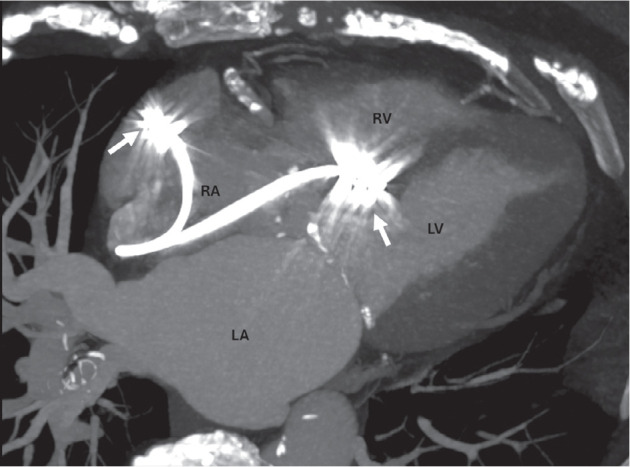
Contrast-enhanced cardiac computed tomography scan with maximal-intensity projection reformation in four-chamber view showing the pacemaker leads located in the right atrium and right ventricular septum (arrows). *Abbreviations:* LA, left atrium; LV, left ventricle; RA, right atrium; RV, right ventricle.

An 80-year-old woman with paroxysmal complete atrioventricular block presented for elective implantation of a dual-chamber pacemaker. The echocardiogram revealed normal ventricular size and systolic function without valvular abnormalities. Her past medical history was significant for hypertension and dyslipidemia. The baseline electrocardiogram (ECG) showed normal sinus rhythm with a normal P–R interval and QRS duration. A stylet-driven lead (5.6-Fr Solia S lead; Biotronik, Berlin, Germany) was introduced through a fixed preformed sheath (Selectra 3D; Biotronik) according to the technique described for LBBAP after signed informed consent was obtained. Beginning from the right ventricle, specifically within the basal septum area of the interventricular septum, a pre-screw septogram was recorded to ensure the perpendicular alignment of the system with the septum. Following this, the lead was cautiously inserted into the septum with five to six clockwise rotations, all the while monitoring changes in QRS morphology. Upon satisfactory observation of the QRS transitioning to LBB-paced morphology **(Figure 1)**, contrast injection was carried out to determine the lead depth. Upon the injection, we observed forward flow from the septum into the coronary venous system, accompanied by a dissecting flap at the lead tip (refer to **Figure 2** and **Video 1**). This site yielded optimal parameters (with a capture threshold of 0.6 V at 0.5 ms and a sensed R-wave of 8 mV). Cardiac shadow motion remained steady, and hemodynamics were stable. The patient denied chest pain or shortness of breath. Subsequently, we withdrew the sheath from the septum and reinjected contrast, but the repeat injection failed to demonstrate contrast penetration into the septal vein. Post-procedure echocardiography confirmed proper lead positioning on the basal septum and the absence of pericardial effusion. A Doppler study did not show abnormal flow in the septal region. Cardiac computed tomography revealed that the lead tip was located within the septum, the lead went through the tricuspid valve and into the right ventricular septum, and there was a non-significant pericardial effusion **(Figure 3)**.

**Video 1:** 

## Discussion

In some instances, LBBAP is performed in cardiac catheterization laboratories without an electrophysiology recording system, relying solely on surface ECG and fluoroscopic guidance.

Perforation of the septal artery was another major concern for this patient. However, in the absence of chest pain, ST-T changes on the ECG, and regional wall motion abnormalities on an echocardiogram, significant septal coronary injury could be definitively excluded, and thus, a left heart catheterization was not warranted.

This uncommon complication has been documented in various instances involving different lead designs and variations in coronary venous anatomy.^2,3^ Special care must be taken to avoid septal perforation, which is considered the most serious complication, along with potential issues such as tricuspid leaflet impingement. Our case presents distinctive characteristics of coronary venous dissection that have not been previously reported. Additionally, the unusually large caliber of the septal vein observed in our case is not commonly documented in existing literature. Evidence supporting the visualization of venous structures includes rapid contrast washout within seconds, which can help rule out contrast filling in the myocardial plane.

A pre-screw septogram has been suggested as a means to prevent coronary venous fistula formation.^1^ However, this approach does not entirely eliminate the risk of coronary venous penetration during lead rotation. Our case highlights the presence of significant variability in this intricate septal anatomy. Despite recommendations from previous reports to reposition the lead, we opted not to do so in our case due to optimal parameters and the absence of further contrast leakage from the right ventricular cavity into the septum. In summary, our study illustrates a unique complication of left bundle branch pacing that can be effectively managed conservatively without the need for lead repositioning.
